# Home and away- the evolutionary dynamics of homing endonucleases

**DOI:** 10.1186/1471-2148-11-324

**Published:** 2011-11-04

**Authors:** Adi Barzel, Uri Obolski, Johann Peter Gogarten, Martin Kupiec, Lilach Hadany

**Affiliations:** 1 Department of Molecular Microbiology and Biotechnology, Tel Aviv University, Ramat Aviv, 69978, Israel; 2 Department of Pediatrics, Stanford University, California, 94305, USA; 3 Department of Molecular Biology and Ecology of Plants, Faculty of Life Sciences. Tel Aviv University, Ramat Aviv, 69978, Israel; 4 Department of Molecular and Cell Biology, University of Connecticut, 91 North Eagleville Road, Storrs, CT 06269-3125 USA

## Abstract

**Background:**

Homing endonucleases (HEases) are a large and diverse group of site-specific DNAases. They reside within self-splicing introns and inteins, and promote their horizontal dissemination. In recent years, HEases have been the focus of extensive research due to their promising potential use in gene targeting procedures for the treatment of genetic diseases and for the genetic engineering of crop, animal models and cell lines.

**Results:**

Using mathematical analysis and computational modeling, we present here a novel account for the evolution and population dynamics of HEase genes (HEGs). We describe HEGs as paradoxical selfish elements whose long-term persistence in a single population relies on low transmission rates and a positive correlation between transmission efficiency and toxicity.

**Conclusion:**

Plausible conditions allow HEGs to sustain at high frequency through long evolutionary periods, with the endonuclease frequency being either at equilibrium or periodically oscillating. The predictions of our model may prove important not only for evolutionary theory but also for gene therapy and bio-engineering applications of HEases.

## Background

Self-splicing introns and inteins are genetic elements that are transcribed as part of genes; they remove themselves from the transcript before translation (introns) or from the translated protein (inteins) [1,2]. Homing endonuclease genes (HEGs) are molecular parasites that are frequently encoded as open reading frames on class I introns, or as part of inteins [3]. The recognition site of these HEases is so large that they cleave a genome only in one or very few places. This has prompted attempts to use HEases in gene therapy and in the genetic engineering of large and complex genomes [4-6]. HEases have been designed to target disease-associated genes, such as XPC [7] and RAG1 [8], to induce targeted integration in cell lines [9,10], and for targeted mutagenesis in crop [11]. In the natural host, a HEase promotes the horizontal propagation of its respective intron/intein into an intron-less or intein-less allele by cleaving the vacant allele to induce double strand break repair by homologous recombination (HR). The finding that the HEases encoded in inteins and introns belong to different endonuclease families [12,13] reveals that the unions between HEGs and self-splicing elements occurred several times, possibly because both HEases and introns/inteins evolved to target similar sites [14]. This union between self-splicing elements and endonucleases created molecular parasites, which have to be regarded as distinct evolutionary units, and whose fate is intertwined but separate from the fate of the host protein and the host organism [15]. The life cycle of inteins/introns with HEGs has been described by the homing cycle [16,17]. The homing cycle addresses the following dilemma: if the HEase is very successful, after a short phase of super-Mendelian inheritance, the HEase targets in all members of the population will be occupied by a HEG; however, once the HEase has ran out of substrate, its activity is no longer under purifying selection; the HEG begins a decay process, resulting in self-splicing elements that lost the ability to home. At the end of this decay process are class I introns that do not encode HEases, and mini inteins, *i.e.*, inteins without a HEase domain. Once the dysfunctional HEG is fixed in the population (through drift or by the selective disadvantage conveyed by the active HEase), the splicing element can be deleted, thus returning the homing cycle to its beginning. In case of introns, the precise deletion may occur via a processed mRNA intermediate [18,19]. Inteins can only be eliminated by random precise deletion, which may be very rare. A precise deletion likely is required to retain a functional host protein, because inteins and class I introns are found in the most conserved parts of conserved proteins, and an imprecise deletion likely would yield a non-functional protein [20].

### Support and discrepancies encountered by the homing cycle model

The homing cycle is based on fixation within and transfer between populations [16,17,21]; however, the homing cycle usually was evaluated on the basis of transfer between species. The transfer of HEG containing inteins and introns across species boundaries is revealed through the high sequence similarity of these molecular parasites found in distantly related organisms [17,22,23]. In many instances, where closely related organisms were sampled, the phylogeny of the HEG was found not to agree with the phylogeny of the host [15,16,24-26]. This confirms the inter-species transfer of HEG containing elements, in agreement with the homing cycle model as formulated for species.

However, some recent findings suggest that HEGs can persist in a single species over very long periods of time. For example, molecular parasites with functional HEGs were found in the apparently asexual amoeba *Naegleria *[27], and the PRP8 intein with HEG persisted over long periods in euascomycetes ([21] provide an estimate of over 500 million years) under purifying selection [28] and without any evidence for their horizontal transfer. Gogarten and Hilario [21] discuss three possible explanations for the long term persistence of HEG containing molecular parasites: (a) the molecular parasites may have acquired a function that provides an adaptive advantage to the host, (b) the homing cycle could operate on a subpopulation level in a spatially distributed inhomogeneous population, (c) the super-Mendelian inheritance may be balanced by selective disadvantages that the HEase and the self-splicing element confer to the host organism. For the latter case Yahara *et al. *[29] showed that stable equilibria can exist, in which all three types of homing sites coexist in a well-mixed population but only under the highly unlikely assumption that the reduction in fitness resulting from carrying a dysfunctional HEase is higher than the reduction in fitness due to the functional HEase. For more realistic conditions, when the cost of the functional HEase is higher than the cost of the dysfunctional HEase, only periodic solutions were reported by [29] that are similar to a typical Lotka-Volterra type predator-prey system [30] with the trajectory following the phases of the homing cycle (invasion, fixation, decay, complete loss; however, the fixation phase does not go to completion, and reinvasion occurs from within the population). Under these conditions and for the parameter choices analyzed by Yahara et al. [29] the average frequency of alleles with functional HEG is low, often verging on extinction during each cycle of the trajectory. This finding is in conflict with the observation that functional HEGs are often sampled from natural populations, suggesting high frequency.

We therefore decided to explore more thoroughly the possibility of long-term persistence of functional HEGs at high frequency. Using analytical and simulation approaches, we study the behavior of an iterative model that simulates allele frequencies of HEGs in randomly interbreeding populations. We report on conditions that allow for the long-term persistence of functional HEGs at high frequency, and we explore the parameter choices under which the persistence of HEGs at high frequency is dynamically stable and evolutionarily stable. We find that, for the range of parameters analyzed, a HEG can persist at high frequency if the effective homing frequency per generation is low, which can be accomplished either through low homing efficiency, or through low frequency of sexual recombination. We also find that an internal equilibrium can be reached with the HEG being at a high frequency, if the self-splicing sequence in which it resides is to some extent toxic. Finally, we find that for these solutions to be evolutionarily stable, in the sense of [31], requires a positive correlation between the homing frequency and the fitness cost of the HEG, an assumption that we discuss and regard as biologically plausible.

**Figure 1:**
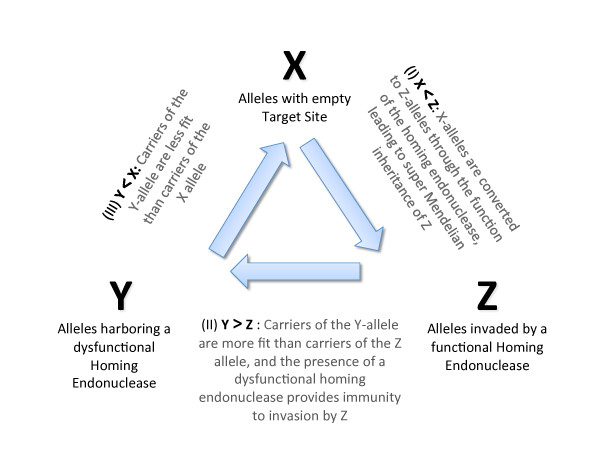
**A Molecular Rock-Paper-Scissors game: The relationships between carriers of the X, Y and Z alleles are descried through three inequalities (I, II, III)**. (I) Z outcompetes X because of the activity of the homing endonuclease during sex (with probability hm), (II) Y beats Z, and (III) X beats Y because of the decrease in relative fitness associated with the homing ability (parameter t) and with the splicing element (parameter s). The central arrows depict the serial succession of the state of a particular homing endonuclease target site: The empty target site (X) is converted to Z through invasion by the homing endonuclease; the homing endonuclease in a Z allele can undergo mutation to become a Y allele with a dysfunctional homing endonuclease, which provides immunity to invasion by Z. Finally, a precise deletion of the intein/intron can restore the empty target site.

We note that the relative fitness of the three alleles in competition represents an incarnation of the rock-paper-scissors game (Figure 1). In pair-wise competition the three alleles form a circular inequality. Given previous explorations of similar non-transitive interaction systems (e.g., [32]), the finding of stable and evolutionarily stable equilibria in a spatially homogenous implementation of the rock-paper-scissors game is surprising and may be relevant for the field of game theory.

## Results

### Model

Our model aims to describe the evolutionary dynamics of HEGs in populations of organisms engaging in genetic exchange. We assume that mating and genetic exchange are random, that generations are discrete and non-overlapping and that selection acts at the haploid stage. We assume the population is large enough to neglect the effect of genetic drift, and allow no gene flow into or out of the population. All organisms in the population are considered isogenic apart from a single gene with three different alleles: X - an allele harboring no insertion sequence; Y - an allele harboring an intron or an intein but no functional HEG; and Z - an allele harboring an intron or an intein encoding for a functional HE. The frequencies of the X, Y and Z alleles are designated x, y and z respectively, with x + y + z = 1.

We assume that a homing endonuclease can degenerate at a rate **u **per generation - converting a Z allele to a Y allele (Additional file 1, Table S1 also showing respective notation in [29] to allow comparison of the two models). The reciprocal conversion is neglected. Unlike the endonuclease activity, a complete degeneration of the splicing function is detrimental for the organism because it impairs the function of the host gene. Modeling the inactivation of the HEG by a single rate constant is a simplification; more subtle changes in either splicing or cleaving efficiency are considered in detail below. The model allows both the Z and the Y alleles to undergo precise elimination at a rate **v **per generation, giving rise to an X allele. The X allele can in turn be converted to a Z allele only through homing. We assume that non-HEase induced gene conversion is globally reciprocal and hence negligible. For the same reason, we allow no X to Y conversion. In our model, **v **is taken to be much smaller than **u**. Precise intron erasure can occur by gene conversion between a genomic Y or Z allele and the cDNA of its transcript [18,19], or through gene duplication through an mRNA intermediate. Complete intein abolition may be rarer because it relies on serendipitous precise deletion. The effect of homing on the Z- and X-allele frequencies is determined by two parameters: **m **- the rate of mating or horizontal gene transfer per generation and **h **- the probability of homing in a diploid carrying an X allele and a Z allele (or the rate of homing in a merodiploid carrying a genomic X allele and an exogenous Z allele). We note that the factors h and m do not occur independently of each other in our equations, and that the product **hm **is a measure of the flux from Z to X being proportional to x*z. Finally, we assume that the insertion sequences may have an effect on the fitness of the organism. We define the relative fitness of an organism carrying an X allele to be **1**. An organism carrying a Y allele has fitness **1-s**, and an organism with a Z allele has fitness **1-s-t**, with **s **and **t **being non-negative and small.

While homing endonucleases are usually considered to be molecular parasites, they may at some point in evolution become beneficial to their host[21,33,34], which would translate to t < 0 in our nomenclature. Clearly, an allele that increases fitness of its carriers can be driven into fixation. A striking example is the HO endonuclease from Saccharomyces cerevisiae which is an evolutionary derivative of a HEG that has acquired an essential role in the yeast mating type switch[35]. During its domestication process, HO has lost its ability for super-Mendelian inheritance. In particular, it is not coded within an intron or an active intein and is not flanked by homologies to its target site. Here we are interested in the evolutionary persistence of homing endonuclease parasites as such. We are interested in the persistence of the homing ability and its linkage to intron/intein mobility. We therefore chose to put aside the case of t < 0 in order to focus on the tri-partite dynamic between two symbiotic parasites (intron and HEG) and their host that takes place for t ≥ 0 values. We define **t **to be the toxicity of the homing activity of the endonuclease, which is assumed to have a non-negative value, as is also assumed for the fitness cost of the insertion sequence (**s**).

We study the above model analytically and numerically to find and describe the parameter sets allowing the evolutionary persistence of homing endonucleases (Z alleles). We derived a set of three recursive functions describing the X, Y and Z allele frequencies at the n+1^th ^generation given their frequencies at the n^th ^generation for a given parameter set **{u, v, h, m, s, t} **(see derivation in additional file 2, Table S2). The symbol W stands for the average fitness of the population W = x + (1-s)y + (1-s-t)z:

(1) $${\text{x}}_{\text{n}+1}=\frac{\;\left[{\text{x}}_{\text{n}}+\text{v}\left({\text{W}}_{\text{n}}-{\text{x}}_{\text{n}}\right)\right]}{{\text{W}}_{\text{n}}}$$

$$\cdot \left[1-\frac{\text{hm}\left(1-\text{v}\right)\left(1-\text{u}\right)\left(1-\text{s}-\text{t}\right){\text{z}}_{\text{n}}}{{\text{W}}_{\text{n}}}\right]$$

(2) $${\text{y}}_{\text{n}+1}=\;\frac{\left(1-\text{v}\right)\left[\left(1-\text{s}\right){\text{y}}_{\text{n}}+\text{u}\left(1-\text{s}-\text{t}\right){\text{z}}_{\text{n}}\right]}{{\text{W}}_{\text{n}}}$$

(3) $${\text{z}}_{\text{n}+1}=1-{\text{x}}_{\text{n}+1}-{\text{y}}_{\text{n}+1}$$

$$\begin{array}{c} \text{W}=\text{x}+\left(1-\text{s}\right)\text{y}+\left(1-\text{s}-\text{t}\right)\text{z}\\ {\text{x}}_{\text{n}+1}=\frac{\left[{\text{x}}_{\text{n}}+\text{v}\left({\text{W}}_{\text{n}}-{\text{x}}_{\text{n}}\right)\right]}{{\text{W}}_{\text{n}}}\\ \cdot \left[1-\frac{\text{hm}\left(1-\text{v}\right)\left(1-\text{u}\right)\left(1-\text{s}-\text{t}\right){\text{z}}_{\text{n}}}{{\text{W}}_{\text{n}}}\right]\\ {\text{y}}_{\text{n}+1}=\frac{\left(1-\text{v}\right)\left[\left(1-\text{s}\right){\text{y}}_{\text{n}}+\text{u}\left(1-\text{s}-\text{t}\right){\text{z}}_{\text{n}}\right]}{{\text{W}}_{\text{n}}}\\ {\text{z}}_{\text{n}+1}=1-{\text{x}}_{\text{n}+1}-{\text{y}}_{\text{n}+1} \end{array}$$

**Figure 2:**
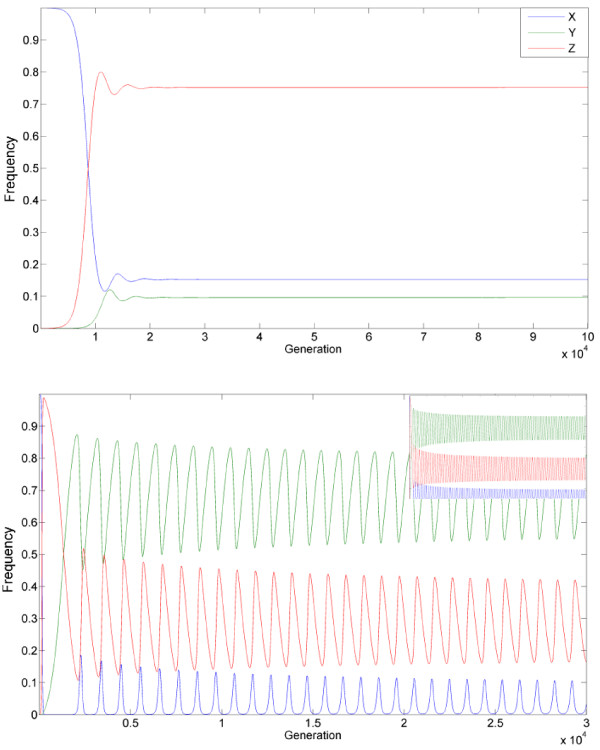
**Examples of homing endonuclease persistence at a high frequency**. **A) **Equilibrium with a high frequency obtained using the parameters: s = 0.01, t = 0.001, hm = 0.0123, u = 10^-4 ^and v = 10^-6^. **B) **A periodic solution obtained using the parameters: s = 0.027, t = 0.0027, hm = 0.0969, u = 10^-4 ^and v = 10^-6 ^(small top panel: same for 10^6 ^generations).

This interplay between the alleles allows the HEG to be maintained in a population, if the frequency of the three alleles reaches either equilibrium (e.g. Figure 2a) or periodic oscillations (e.g. Figure 2b). Notably, when equilibrium is reached, it is often reached as the damping of oscillations, and in finite populations it is sensitive to stochastic perturbations to an extent discussed below.

In contrast to previous reports [29], we find a plausible range of parameters that allows all three alleles to be maintained at an internal equilibrium with the HEG (Z allele) being at high frequency (e.g. Figure 2A). The conditions and inter-dependencies between the parameters that define this range can be discerned from an analysis of the equilibrium equations: x_n+1 _**= **x_n _and, y_n+1 _**= **y_n _(and therefore also z_n+1 _**= **z_n_**)**. The analysis leads to some non-trivial, perhaps even surprising, conclusions demonstrating the uniqueness of the evolutionary dynamics of homing endonucleases.

### Analytical bound 1

For an internal equilibrium to be maintained with the Z allele being at high frequency, both the Y and the Z alleles must have a reduced fitness with respect to the X allele. In particular, because the parameter s must be larger than or equal to a non-negligible positive number:

$\frac{\text{u}\left(\text{1}-\text{t}\right)\left(\text{1}-\text{v}\right)-\text{v}\frac{{\text{y}}_{\text{eq}}}{{\text{x}}_{\text{eq}}}}{\left(\text{1}-\text{v}\right)\left(\frac{{\text{y}}_{\text{eq}}}{{\text{x}}_{\text{eq}}}+\text{u}\right)}$. In other words, the insertion sequence must be toxic in order for the HEG to reach high equilibrium frequency.

### Mathematical formulation

(4) $$\frac{{\text{y}}_{\text{eq}}}{{\text{x}}_{\text{eq}}}>0\;\Rightarrow \text{s}\geq \frac{\text{u}\left(\text{1}-\text{t}\right)\left(\text{1}-\text{v}\right)-\text{v}\frac{{\text{y}}_{\text{eq}}}{{\text{x}}_{\text{eq}}}}{\left(\text{1}-\text{v}\right)\left(\frac{{\text{y}}_{\text{eq}}}{{\text{x}}_{\text{eq}}}+\text{u}\right)}$$

(see additional file 3, Proof S1).

In particular, ${\text{z}}_{\text{eq}}>{\text{y}}_{\text{eq}}\Rightarrow \text{s }\geq \frac{\text{u}\left(\text{1}-\text{t}\right)\left(\text{1}-\text{v}\right)-\text{v}}{\left(\text{1}-\text{v}\right)\left(\text{1}+\text{u}\right)}$. We note that the HEG degeneration rate (u) is expected to be orders of magnitude larger than the introns/intein deletion.

### Analytical bound 2

For an internal equilibrium to be maintained with the Z allele being at a high frequency,, the reduction in fitness associated with the endonuclease activity of the Z allele (t) has to be smaller than the reduction in fitness associated with the splicing element (s) or of similar magnitude to it. In particular, the parameter t must be smaller than or equal to the positive number: $\frac{\text{v}+\left(\text{1}-{\text{z}}_{\text{eq}}\right)\text{s}}{{\text{z}}_{\text{eq}}}$. That is, the endonuclease must be precise to the extent that its activity is not significantly more toxic than the insertion sequence per se.

### Mathematical formulation

(5) $${\text{z}}_{\text{eq}}>\text{0}\Rightarrow \text{t }\leq \frac{\text{v}+\left(\text{1}-{\text{z}}_{\text{eq}}\right)\text{s}}{{\text{z}}_{\text{eq}}}$$

(see additional file 4, Proof S2).

E.g. z_eq _> 0.5 ⇒ t ≤ s + 2v.

### Analytical bound 3

For an internal equilibrium to be maintained with the Z allele being at high frequency, the transmission rate of the HEG must be low (albeit, above a positive threshold). In particular, the product hm must be smaller than or equal to the small number: $\frac{\text{t}+\frac{\left(\text{s}+\text{2}\frac{\text{v}}{\text{u}}\right)}{{\text{z}}_{\text{eq}}}}{\left(\text{1}-\text{v}\right)\left(\text{1}-\text{u}\right)\left(\text{1}-\text{s}-\text{t}\right)}$,. We note that selection of various Z alleles in the presence of empty target sites (i.e. high x) would favor high homing ability, at least until specificity is optimal and any further increase in homing ability is accompanied by increased toxicity (see below). Therefore, we would expect to find homing endonucleases persisting at high equilibrium levels only in organisms characterized by low rates of genetic exchange (low mating and or low recombination rates).

### Mathematical formulation

(6) $${\text{z}}_{\text{eq}}>\text{0}\Rightarrow$$

$$\frac{\left(\text{s}+\text{t}\right)}{\left(\text{1}-\text{u}\right)\left(\text{1}-\text{v}\right)}<\text{hm }$$

$$\text{hm }\leq \frac{\text{t}+\frac{\left(\text{s}+\text{2}\frac{\text{v}}{\text{u}}\right)}{{\text{z}}_{\text{eq}}}}{\left(\text{1}-\text{v}\right)\left(\text{1}-\text{u}\right)\left(\text{1}-\text{s}-\text{t}\right)}$$

(See additional file 5, Proof S3).

In particular, ${\text{z}}_{\text{eq}}>\frac{1}{2}\Rightarrow$.

$$\frac{\left(\text{s}+\text{t}\right)}{\left(\text{1}-\text{u}\right)\left(\text{1}-\text{v}\right)}\;<\text{ hm}$$

$$\text{hm }\leq \frac{\text{t}+\left(\text{2s}+\text{4}\frac{\text{v}}{\text{u}}\right)}{\left(\text{1}-\text{v}\right)\left(\text{1}-\text{u}\right)\left(\text{1}-\text{s}-\text{t}\right)}$$

### Comparison with the Yahara model

We and Yahara et al. [29] have used similar models with similar goals and reached strikingly different conclusions. Yahara et al. found stable internal equilibria to be possible only under the condition that the decrease in fitness resulting from carrying a dysfunctional HEase is higher than the decrease in fitness due to the functional HEase. Otherwise, they have identified only periodic solutions. In contrast, we found a significant and biologically plausible range of parameters allowing for stable equilibria to be maintained, all under the assumption that functional HEase decreases fitness more than dysfunctional HEase (namely, 0 < t). When we take a parameter set leading to stable equilibrium in our model and input it into the Yahara model we do find a stable equilibrium there as well (e.g. additional file 6, Figure S1). We assume that Yahara et al. were unable to detect the parameter range allowing for equilibrium because they only included mutation in their calculations when looking for equilibrium with z = 1. When searching for internal equilibria, Yahara et al. acted under the explicit assumption that mutation was a destabilizing factor, therefore neglecting it altogether. In contrast, we have shown that a positive mutation rate can facilitate the establishment of a stable equilibrium. Moreover, the Z equilibrium frequency in Additional file 6, Figure S1 is low (z ≈ 0.092) because the parameter choice adheres to the Yahara restriction (1-s)^2 ^= 1-s-t (*β*^2 ^= α in their notation). When we allow s and t to be independent, stable internal equilibria with high Z frequency are readily detected (See Additional file 7, Figure S2). In summary, the difference between the conclusions made by Yahara and us does not result from model differences (see Additional file 1, Table S1), but rather from the fact that Yahara et al. did not explore the parameter space exhaustively.

### Computer simulation

**Figure 3:**
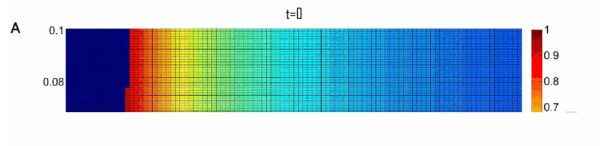
**Phase diagrams depicting the evolutionary fate of the Z allele, in color scale (Right), as a function of its homing ability and toxicity**. The colors correspond to the average Z frequency in the last 10^4 ^of 10^6 ^generations, u = 10^-4 ^and v = 10^-6 ^for all subfigures. A is for t = 0, B t = 0.1 s, and C t = s. When our deterministic simulations reached equilibrium we tested the asymptotic stability of that equilibrium by evaluating the eigenvalues of the Jacobian matrix at that point. The black line separates parameter values allowing for stable equilibria (bottom) from parameter values leading to no equilibria or non stable equilibria.

To further elucidate the conditions facilitating the preservation of the Z allele we carried out a systematic sampling of the parameter space. For each parameter set we used a deterministic computer simulation starting with an initial population consisting almost entirely of X alleles, a miniscule proportion of Z alleles (10^-4^) and no Y alleles. These starting conditions best model actual populations being invaded by HEGs. We stopped the simulation once equilibrium of allele frequencies has been reached. Equilibrium was assumed if the last 10,000 generations had a standard deviation, which was no more than 10^-5^. Additionally, if frequency of the HEG went under 0.001 after having been over 0.01, the frequency of z was given the value zero (to avoid cases which are likely to be stochastically eliminated in a finite population). In case no stable equilibrium was reached, we plot the average frequency of Z in the last 10^4 ^of 10^6 ^generations. Our results are presented as phase diagrams depicting the evolutionary fate of the Z allele on color scale as a function of its homing ability and toxicity (e.g. Figure 3). For each phase diagram we sampled 10^4 ^different parameter sets by crossing 100 different choices for the value of the product hm with 100 different choices for the value of s. Different relations between s and t were selected for each phase diagram as indicated, while u = 10^-4 ^and v = 10^-6 ^were taken to be constant and equal in all phase diagrams.

The sub-space of parameter choices leading to stable allele frequency equilibria is bordered in our phase diagrams by a solid black line (Figure 3). Importantly, the phase diagrams demonstrate that analytical bounds 1-3, allowing high equilibrium frequency of the Z allele, are tight, i.e. for almost any parameter set obeying these conditions, the homing endonuclease will indeed reach a high equilibrium frequency. Moreover, the conditions allowing the Z allele to reach a high average frequency in a limit cycle are a natural extension of the conditions allowing it to have high equilibrium frequency:

1. Analytical bound 1 states that the insertion sequence must be detrimental (s > 0) for an internal equilibrium to be reached where the Z allele has a high frequency. Our phase diagrams illustrate that s > 0 is furthermore a prerequisite for the Z allele to reach a high average frequency. This condition is biologically plausible. Even if the intein or intron has optimized its splicing activity, the insertion sequence still bears with it the burden of replicating, transcribing and in the case of inteins also translating, hundreds of superfluous nucleotides.

2. Analytical bound 2 states that the endonuclease activity cannot be significantly more toxic than the insertion sequence per se (t < ≈ s) for an internal equilibrium to be reached where the Z allele has a high frequency. Our phase diagrams demonstrate a clear trend for higher average z values to be associated with a lower t/s ratio (compare Figure 3a, b and 3c).

3. Analytical bound 3 states that an internal equilibrium with a high frequency of z is possible only when the product hm is low, but not too low, so that hm > ≈ s+t. For this reason, the values of hm are presented in the phase diagrams as products of s+t. In congruence with analytical bound 3, our phase diagrams demonstrate that high average frequencies of the Z allele are possible only when s + t < ≈hm < 2(s+t). Low rates of genetic exchange are therefore necessary for long term HEG persistence.

### Model predictions for finite populations

**Figure 4:**
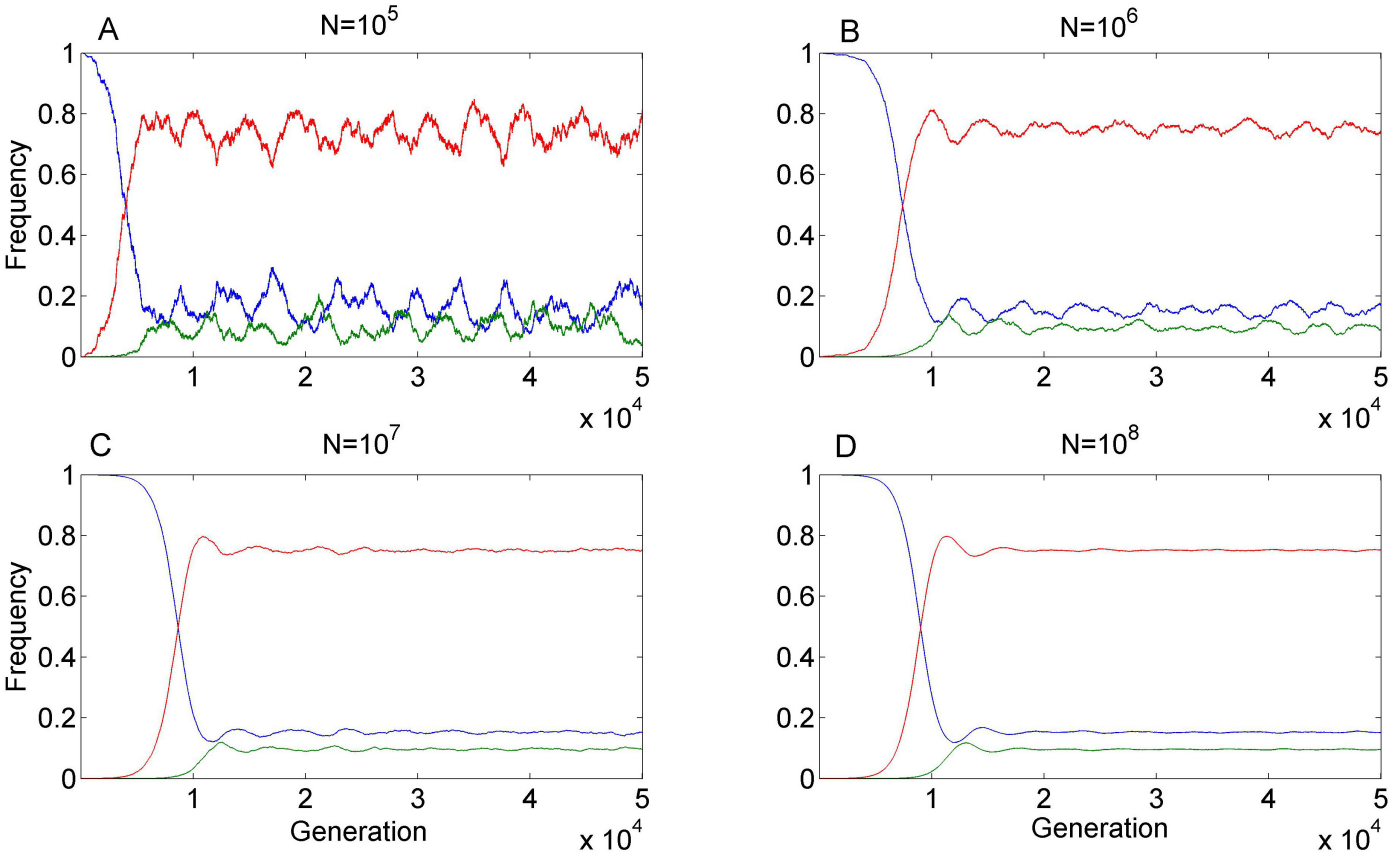
**Example of model predictions for finite populations**. Random noise was added to our deterministic model as expected from binomial sampling in a population of size N = 10^6^,10^7 ^or 10^8 ^(A-C, respectively). The parameters used: s = 0.01, t = 0.001, hm = 0.0123, u = 10^-4 ^and v = 10^-6^, are identical to those in Figure 2a and result in a stable equilibrium in the deterministic model.

Our analysis so far assumed an infinite population. For finite populations, genetic drift may delay, restrict or preclude the damping of the initial oscillatory dynamic (e.g. Figure 4 and Additional file 8, Figure S3). When the absolute values of the eigenvalues of the Jacobian are smaller than 1 but the eigenvalues do have imaginary parts (as is typically the case in our stable area), a deterministic system approaches equilibrium through oscillations (e.g. Figure 2), and a finite population would exhibit some oscillations around equilibrium. When the absolute value of the complex eigenvalue is very close to 1, the oscillations decay slowly. Then the question is the rate of oscillations decay versus the strength of the perturbations resulting from random sampling. In particular, when the population is relatively small, so that drift results in significant perturbations, the system does not converge to equilibrium and sustains oscillations (e.g. Additional file 8, Figure S3 A). In larger populations convergence to near equilibrium does happen (e.g. Figure 4 and Additional file 8, Figure S3 B-D). This finding is in congruence with the abundance of HEGs in unicellular organisms, which have a larger characteristic population size (≥ 10^7 ^[36-39]).

### Evolutionary stability

The dynamic stability of an allele frequency equilibrium is not sufficient to guarantee the long term persistence of a HEG in a real population [31]. This is because alleles with different homing ability and different toxicity do arise by mutation, threatening to disturb any status quo. Therefore, we set out to explore the stability of equilibria with high z values with respect to invasions by alleles with different attributes in order to discern the conditions rendering a homing endonuclease evolutionarily stable.

### 1. Invasions by Z' alleles with different homing ability (h') and/or different homing related toxicity (t')

**Figure 5:**
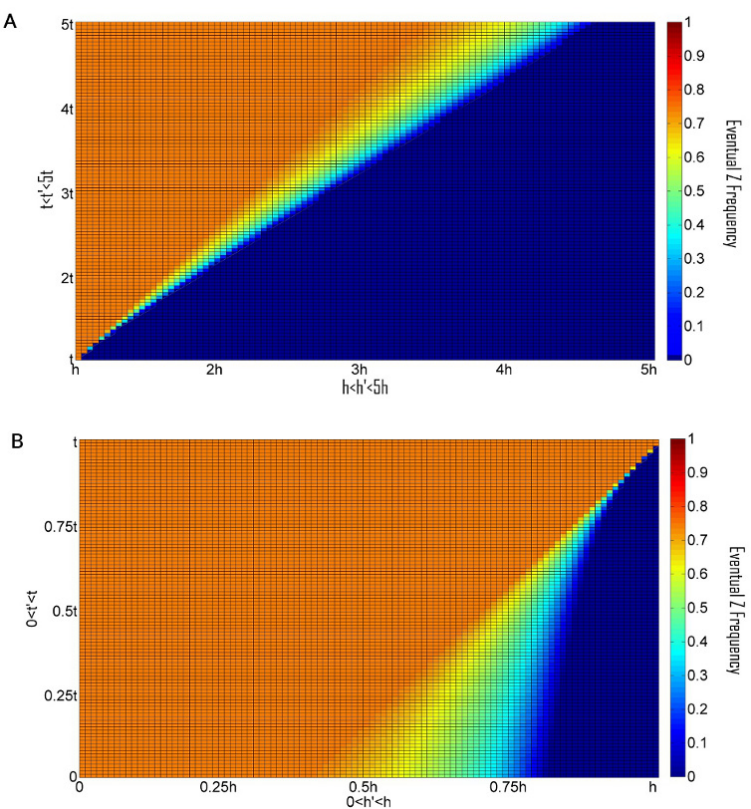
**Phase diagrams depicting the evolutionary fate of the Z allele, in color scale (Right), after an invasion by a Z' allele, with a different homing ability h' and toxicity t'**. A. h' < h and t' < t. B. h' > h and t' > t. Expectedly, for h' < h and t' > t, Z' will never invade, while for h' > h and t' < t, Z' will always invade (results not shown). The colors correspond to the average Z frequency in the last 10^4 ^of 10^6 ^generations, u = 10^-4^, v = 10^-6^, and the rest of the parameters were chosen for a high value of z (0.733) in a stable equilibrium: s = 10^-2^, t = 10^-3^, hm = 0.0123.

All other parameters being equal, any mutation in a Z allele giving rise to a Z' allele having a higher specificity should prevail (i.e., any Z' allele with (h' > h and t' ≤ t) OR (h' ≥ h and t' < t))). A particular Z' may have a higher equilibrium value or a higher oscillation average than did Z, but if such invasions of Z alleles with higher specificity are repeated, eventually they might lead to the fixation of the HEG allele and to its subsequent degeneration. This destructive scenario is avoided, if we assume that when maximal specificity is obtained then the homing ability is at its biological upper bound and the toxicity is at its biological lower bound. Evolutionary stability of Z is made possible for a broader range of h and t values under the biologically plausible assumption that once a threshold specificity is obtained, (A) any possible rise in potency "h" is accompanied by a rise in toxicity "t"; and vice versa: (B) any possible decrease in t is accompanied by a decrease in h (Figure 5A and 5B). We note that conditions A) and B) may conflict for moderate values of h and t. Therefore, we expect to find internal equilibria where the Z allele has a high frequency, if the toxicity or homing ability of the HEG is near the respective biological bounds.

### 2. Invasions by Y' or Z' alleles with lower intron/intein related toxicity (s')

A mutation in a Y allele can give rise to a Y' allele with a lower toxicity s' < s. However Y' may not invade because Y is constantly regenerated by HEG degeneration. On the other hand, if the mutation reducing s to s' takes place in a Z allele (a mutation in the splicing motif of the HEG containing intron/intein) then the resulting Z' allele and its degenerated form Y' are expected to win over Z and Y respectively. As before, Z' may have a higher equilibrium value or higher oscillation average than did Z, but if such invasions are allowed to repeat till s = 0 they will eventually lead to the fixation of a HEG allele and to its subsequent degeneration. Therefore, allele equilibrium with a high Z frequency is evolutionarily stable only if the s parameter is at its physical lower bound, and only if the order of magnitude of this lower bound is not in itself lower than the order of magnitude of u. Again, this is biologically plausible. The insertion sequence still bears with it the burden of replicating, transcribing and, in the case of inteins, also translating, hundreds of superfluous base-pairs.

### 3. Invasions by an X allele with lower homing susceptibility

**Figure 6:**
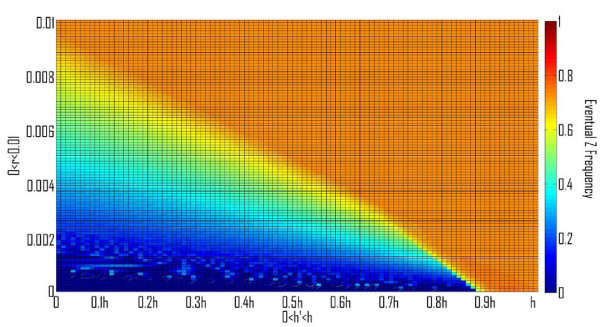
**A phase diagram depicting the evolutionary fate of the Z allele, in color scale (Right), after an invasion by X' allele, a host sequence to which homing is more difficult, as a function of the parameters of X': the reduced homing ability (h') and the cost to the host (r)**. The colors correspond to the average Z frequency in the last 10^4 ^of 10^6 ^generations, u = 10^-4^, v = 10^-6^, and the rest of the parameters were chosen for a high value of z (0.733) in a stable equilibrium: s = 10^-2^, t = 10^-3^, h = 0.0123.

From the host perspective, evading the HEG parasite might carry a fitness benefit. A mutation in an X allele occurring at the target site of the endonuclease can give rise to an X' allele with respect to which the Z allele has a reduced homing ability (h'). Under some circumstances, an invasion of this type may even increase the ultimate frequency of Z by preventing its fixation. However, if such invasions are repeated they will eventually lead to the product hm being lower than $\frac{\text{s}+\text{t}}{\left(\text{1}-\text{u}\right)\left(\text{1}-\text{v}\right)}$, precluding the establishment of any equilibrium with z > 0 (analytical bound 3). To secure the long term stability of the HEG, any reduction in the susceptibility of the empty allele for homing must be accompanied by a large enough reduction in the fitness of the empty allele (h' < h ⇒ fitness of X = 1-r' where r' > 0) (Figure 6). This idea has valid biological support: Type I introns and inteins in general, and with HEGs in particular, are almost always found inserted in conserved sites of essential genes [40]. As a result of this pivotal location, if the host tries to evade the homing endonuclease parasite by changing nucleotides at the target site, then the host has to pay the cost associated with altering a highly conserved motif. Recently, in relation to HEG usage in gene therapy, we showed that HEG target conservation allows some HEases to specifically cleave human loci of medical interest [5].

## Discussion

### Support for the model

At least in some HEG families, HEGs have persisted over long evolutionary periods (up to 500 million years [21]) under purifying selection and without any evidence for their horizontal transfer [27,28]. This is in conflict with the idea of frequent HEG elimination and re-invasion [16,17] and also in conflict with the notion of oscillating HEG frequency nearing extinction at every cycle [29]. It is instead suggestive of a balancing evolutionary mechanism, keeping the HEG-containing allele at high frequency although it is usually associated with no fitness advantage for the host. Direct evidence for our rock-paper-scissors model is scarce. However, some indirect data imply that our predictions are valid. Vos and Didelot [41] compared recombination rates (Corresponding roughly to the hm values in our model) in varied bacterial and archaeal species. All marine bacteria surveyed (*M. aeruginosa, M. chthonoplastes, P. shigelloides, P. ubique, V. parahaemolyticus, V. vulnificus*) were characterized by high recombination rates with the exception of *Microcoleus chtonoplastes*. *M. chtonoplastes *is also the only one of these species known to encode a HEG (in the RIR1-2 intein). Amongst Eukaryotes, yeasts are particularly abundant with HEGs. Several studies indicate that sex is yeast is indeed rare (as low as 1 out-crossing event in 50,000 generations in *Saccharomyces cerevisiae *and *Saccharomyces paradoxus *[42] and as low as 1 sexual cycle in 1000 generations in *S. paradoxus *[37]) Finally, HEGs are widespread in mitochondria and chloroplasts. Both of these endosymbionts are notorious for their low rates of genetic exchange [43,44].

Our analysis of finite populations exemplified how genetic drift can sometime prevent the stabilization of allele-frequency oscillations, endangering the long term persistence of the HEG. The phenomenon is especially pronounce in populations of smaller size, which may in part explain the prevalence of HEG in unicellular organisms characterized by large population sizes[36-39].

Our prediction that some toxicity of the insertion sequence is inevitable is supported by the positive correlation found between gene compactness and gene expression [45-47]. We further predict that for long term HEG persistence at high levels the toxicity of the endonuclease must not be higher than the toxicity of the insertion sequence per se. Indeed, HEases were shown to possess exquisite specificity. For example, the I-SceI HEase from the mitochondria of the budding yeast *Sacharomyces cerevisiae *(genome size ~8.5*10^4 ^bp) has an extremely narrow and predictable range of cleavage sites in the human genome [48] (genome size ~3*10^9 ^bp) and little if any toxicity in human cells and live mice [6,49]. Last, the importance and hence the conservation of intron and intein insertion sites, as predicted by our model, have been repeatedly documented [20].

Finally, according to our model, certain HEGs might be stable in the short term but not in the long term, in the sense that particular mutants that might lead to extinction of the HEG can invade the population. In these cases the long term pattern of HEG evolution might be that of the homing cycle [21], but orders of magnitude slower.

### Possible applications for the predictions of the model

It is interesting to assess our model not only from the perspective of evolutionary biology, but also in terms of the relevance of its predictions to gene therapy and genetic engineering.

HEases may have very long target sites [3]. However, given the degeneracy in target recognition [50], the question remains whether many HEases, selected to cleave small genomes of microbes, mitochondria and chloroplasts would turn out to have a multitude of targets in the human genome and therefore would be highly toxic. While most parasites (e.g. viruses) can balance their toxicity with their ability to propagate and therefore could bear significant toxicity to their natural host, the same is not true for HEases. The Rock-Paper-Scissors model we put forward asserts that the toxicity of the HEase cannot be balanced by its ability to propagate but that in fact this toxicity is bound by the toxicity of the intron or intein (see analytical bound 2). We therefore expect the toxicity of the HEG to be extremely low in its natural host, possibly implying reduced toxicity in human cells.

Finally, our analysis of the evolutionary stability of HEG alleles in the face of X' invasions is of importance for future applications of HEases. We asserted that Z can persist only if the reduction in homing ability associated with the X' allele is accompanied by a substantial fitness cost. In other words, a HEase must base its target specificity on conserved base pairs that are crucial for the host. The HEase target specificity, being the range of nucleotide sequences it can cleave, is highly predictable based on knowledge of the degree of conservation of individual sites along the target [24,51-54]. Furthermore, Because HEGs reside within introns or inteins found in conserved motifs, finding a HEase target in the human genome may often not be a random event but rather a result of evolutionary motif conservation from the HEG hosting microbe to humans[5].

Our model of HEG population dynamics delineates the under-recognized complexity of the evolution of selfish elements. A better understanding of these fascinating molecular parasites could pave the way for their wide application in gene therapy and bio-engineering.

## Conclusions

We provide for the first time a plausible account for the evolutionary preservation of HEGs. Admittedly, our model is a simplification in that it assumes a large homogenous mixed population, non-overlapping generations, selection acting only at the haploid stage and more. However, even under these simplified conditions, we find solutions in which the active HEG can persist at high frequency over long periods of time. This is in agreement with the fact that HEGs are readily sampled from natural populations of varied organisms [3]. Furthermore, our analysis delineates the conditions allowing long term HEG persistence. In particular we find that long-lasting high frequencies of the Z allele are possible only if the population is characterized by low rates of genetic exchange, if the insertion sequence is to some extent toxic, if the endonuclease activity is not significantly more toxic than the insertion sequence per se, and if the target site is important for the survival of the host.

When the first indications for long-term persistence of functional HEGs in populations were uncovered, one explanation was to assume spatially distributed, inhomogeneous populations, as was suggested for other biological non-transitive competition networks [32,55]. Here we have shown that, perhaps surprisingly, spatial inhomogeneity is not a prerequisite for long-term persistence of HEGs at high penetrance. We determined conditions that are compatible with long-term HEG persistence, and we find that these conditions are met in populations that show a high frequency of HEGs.

## List of abbreviations

HEase: Homing Endonuclease; HEG: Homing Endonuclease Gene; HGT: Horizontal Gene Transfer.

## Authors' contributions

AB Conceived of the study, designed the model and performed the mathematical analysis, UO designed, preformed and analyzed the computer simulations, JPG participated in the design of the study and in data analysis, MK participated in the design of the study and in data analysis, LH coordinated the study, participated in the design of the study and in data analysis. All authors read and approved the final manuscript.

## Supplementary Material

Additional file 1 **Table S1 - Parameter index**. A table containing an index of all parameters being used as well as a comparison to the Notation in Yahara et al[29]. Click here for file

Additional file 2 **Table S2 - Derivation of recursive functions describing the dynamics of allele distribution**. A table containing the derivation of the recursive functions describing the dynamics of allele distribution. Click here for file

Additional file 3 **Proof S1 - Proof of analytical bound 1**. Proof of analytical bound 1. Click here for file

Additional file 4 **Proof S2 - Proof of analytical bound 2**. Proof of analytical bound 2. Click here for file

Additional file 5 **Proof S3 - Proof of analytical bound 3**. Proof of analytical bound 3. Click here for file

Additional file 6 **Figure S1 - Examples of Homing endonuclease persistence in Yahara's model**. Examples of Homing endonuclease persistence in Yahara's model obtained using the parameters: α = 0.989, $\beta =\sqrt{\alpha }$, r = 0.0123, u = 10^-4^, v = 10^-6 ^and the initial frequency (in Yahara's notation): x = 0.9999, y = 10^-4^, z = 0. Iterating it for one million generations in Yahara's model, we get a stable equilibrium (determined using numeric computations of the eigenvalues) with frequencies X ≅ 0.51, y ≅ 0.41, z ≅ 0.081. Click here for file

Additional file 7 **Figure S2 - Examples of Homing endonuclease persistence at a high frequency**. Examples of Homing endonuclease persistence at a high frequency in both ours and Yahara's models. Initial frequencies are the same as in Additional file 6. A) Stable equilibrium, using our model, with final z frequency of 0.314 obtained using the parameters: s = 0.01, t = 0.001, hm = 0.0323, u = 10^-4 ^and v = 10^-6^. B) Stable equilibrium, using Yahara's model, with final z frequency of 0.655 obtained using parameters that are analogous the the parameters in A (as explained in Additional file 1, Table S1). Click here for file

Additional file 8 **Figure S3 - Example of model predictions for finite populations**. Example of model predictions for finite populations. Random noise was added to our deterministic model as expected from binomial sampling in a population of size *N *= 10^5^, 10^6^, 10^7^, 10^8 ^(A-D, respectively). The parameters used result in a stable equilibrium in the deterministic model: s = 0.01, t = 0.001, hm = 0.0165, u = 10^-4 ^and v = 10^-6^. Click here for file
