# Supplementary material for: Home and away- the evolutionary dynamics of homing endonucleases
Source: BMC Evol Biol. 2011 Nov 4;11:324. doi: 10.1186/1471-2148-11-324 (PMC3229294; doi:10.1186/1471-2148-11-324)
Supplement: Additional file 1 — Table S1 - Parameter index. A table containing an index of all parameters being used as well as a comparison to the Notation in Yahara et al[29]. [file 1471-2148-11-324-S1.DOCX]

**Additional file 1**

**Table S1- Parameter index**

| Parameter Meaning | Notation in our model | Notation in Yahara's model (29) |
| --- | --- | --- |
| Relative fecundity of an organism carrying an allele harboring an intron or an intein encoding for a functional homing endonuclease. | 1-s-t | α |
| Relative fecundity of an organism carrying an allele harboring an intron or an intein encoding for but no functional homing endonuclease. | 1-s | β |
| Probability of degenerating from functional to non functional homing endonuclease | u | u |
| Probability of precise deletion of non functional homing endonuclease | v | v |
| Probability of precise deletion of functional homing endonuclease | v | - |
| Rate of successful homing | hm | r |
