# Supplementary material for: Home and away- the evolutionary dynamics of homing endonucleases
Source: BMC Evol Biol. 2011 Nov 4;11:324. doi: 10.1186/1471-2148-11-324 (PMC3229294; doi:10.1186/1471-2148-11-324)
Supplement: Additional file 2 — Table S2 - Derivation of recursive functions describing the dynamics of allele distribution. A table containing the derivation of the recursive functions describing the dynamics of allele distribution. [file 1471-2148-11-324-S2.DOCX]

**Additional file 2**

**Table S2- Derivation of recursive functions describing the dynamics of allele distribution**

|  | **X** | **Y** | **Z** |
| --- | --- | --- | --- |
| Frequency distribution at generation n | $x_{n}$ | **$y_{n}$** | $z_{n} \left(=1-x_{n}-y_{n}\right)$ |
| Selection | **$\frac{x_{n}}{W_{n}}$** | **$\frac{\left(1-s\right)y_{n}}{W_{n}}$** | $\frac{ \left(1-s-t\right)z_{n}}{W_{n}}$ |
| Mutation | $\frac{x_{n}}{W_{n}}$ | **$\frac{\left(1-s\right)y_{n}+u\left(1-s-t\right)z_{n}}{W_{n}}$** | **$\frac{ \left(1-u\right)\left(1-s-t\right)z_{n}}{W_{n}}$** |
| Precise deletion | $\frac{x_{n}+v\left(W_{n}-x_{n}\right)}{W_{n}}$ | **$\frac{\left(1-v\right)}{W_{n}}\left[\left(1-s\right)y_{n}+u\left(1-s-t\right)z_{n}\right]$** | **$\frac{ \left(1-v\right)\left(1-u\right)\left(1-s-t\right)z_{n}}{W_{n}}$** |
| Mating/HGT and homing | $\begin{matrix} \frac{x_{n}+v\left(W_{n}-x_{n}\right)}{W_{n}} \\ -hm\left(1-v\right)\left(1-u\right)\left(1-s-t\right) \\ \cdot\frac{z_{n}}{W_{n}^{2}}\left[x_{n}+v\left(W_{n}-x_{n}\right)\right] \end{matrix}$ | **$\frac{\left(1-v\right)}{W_{n}}\left[\left(1-s\right)y_{n}+u\left(1-s-t\right)z_{n}\right]$** | $\begin{matrix} \frac{ \left(1-v\right)\left(1-u\right)\left(1-s-t\right)z_{n}}{W_{n}} \\ +hm\left(1-v\right)\left(1-u\right)\left(1-s-t\right)z_{n} \\ \cdot\frac{\left[x_{n}+v\left(W_{n}-x_{n}\right)\right]}{W_{n}^{2}} \end{matrix}$ |
| Frequency distribution at generation n+1 | $\begin{matrix} x_{n+1}=\frac{\left[x_{n}+v\left(W_{n}-x_{n}\right)\right]}{W_{n}} \\ \cdot\left[1-\frac{hm\left(1-v\right)\left(1-u\right)\left(1-s-t\right)z_{n}}{W_{n}}\right] \end{matrix}$ | **$\begin{matrix} y_{n+1}= \\ \frac{\left(1-v\right)}{W_{n}}\left[\left(1-s\right)y_{n}+u\left(1-s-t\right)z_{n}\right] \end{matrix}$** | $\begin{matrix} z_{n+1}=\left(1-v\right)\left(1-u\right)\left(1-s-t\right)\frac{ z_{n}}{W_{n}} \\ \cdot\left(1+\frac{hm\left[x_{n}+v\left(W_{n}-x_{n}\right)\right]}{W_{n}}\right) \\ \end{matrix}$ |
