# Supplementary material for: Home and away- the evolutionary dynamics of homing endonucleases
Source: BMC Evol Biol. 2011 Nov 4;11:324. doi: 10.1186/1471-2148-11-324 (PMC3229294; doi:10.1186/1471-2148-11-324)
Supplement: Additional file 3 — Proof S1 - Proof of analytical bound 1. Proof of analytical bound 1. [file 1471-2148-11-324-S3.DOCX]

**Additional file 3:**

Proof S1- Proof of analytical bound 1

Theorem 1: **$\forall\text{0<a, az}_{\text{eq}}>\text{y}_{\text{eq}}\Rightarrow\text{s }>\text{ }\frac{\text{u}\left(1-\text{t}\right)\left(1-\text{v}\right)-\text{av}}{\left(1-\text{v}\right)\left(\text{a}+\text{u}\right)}$**

Proof:

1. At equilibrium: **$\text{y }=\text{ }\left(1-\text{v}\right)\left[\frac{\left(1-\text{s}\right)\text{y}}{\text{W}}+\frac{\text{u}\left(1-\text{s}-\text{t}\right)\text{z}}{\text{W}}\right]$**
2. **$\text{yW }=\text{ }\left(1-\text{v}\right)\left[\left(1-\text{s}\right)\text{y}+\text{u}\left(1-\text{s}-\text{t}\right)\text{z}\right]$**
3. **$\text{u}\left(1-\text{s}-\text{t}\right)\left(1-\text{v}\right)\text{z }=\text{ y}\left[\text{W}-\left(1-\text{v}\right)\left(1-\text{s}\right)\right]$**
4. It is biologically true that: **$\text{u}>0,\text{ s}+\text{t}<1,\text{ v}<1$**.
5. **$\text{y}=0\Rightarrow\text{z}=0$** in contradiction to the assumption **$\text{az}_{\text{eq}}>\text{y}_{\text{eq}}$**. Therefore y>0.
6. **$\frac{\text{z}}{\text{y}}\text{ }=\text{ }\frac{\text{W}-\left(1-\text{v}\right)\left(1-\text{s}\right)}{\text{u}\left(1-\text{s}-\text{t}\right)\left(1-\text{v}\right)}$**
7. **$\frac{az}{y}=\frac{a\left[W-(1-v)(1-s)\right]}{u(1-s-t)(1-v)}$**
8. **$\begin{matrix} \text{az}_{\text{eq}}>\text{y}_{\text{eq}}\Rightarrow\\ \text{a}\left[\text{W}-\left(1-\text{v}\right)\left(1-\text{s}\right)\right]\text{ }>\text{ u}\left(1-\text{s}-\text{t}\right)\left(1-\text{v}\right) \end{matrix}$**
9. **$\text{a}\left[1-\left(1-\text{v}\right)\left(1-\text{s}\right)\right]\text{ }>\text{ u}\left(1-\text{s}-\text{t}\right)\left(1-\text{v}\right)$**
10. **$\text{a}\left[\text{v}+\text{s}\left(1-\text{v}\right)\right]\text{ }>\text{ u}\left(\text{1 }-\text{t}\right)\left(1-\text{v}\right)-\text{su}\left(1-\text{v}\right)$**
11. **$\left(\text{a}+\text{u}\right)\left(1-\text{v}\right)\text{s }>\text{ u}\left(1-\text{t}\right)\left(1-\text{v}\right)-\text{av}$**
12. **$\text{az}_{\text{eq}}>\text{y}_{\text{eq}}\Rightarrow\text{s }>\text{ }\frac{\text{u}\left(1-\text{t}\right)\left(1-\text{v}\right)-\text{av}}{\left(1-\text{v}\right)\left(\text{a}+\text{u}\right)}$** **QED**
13. In particular **$\text{z}_{\text{eq}}>\text{y}_{\text{eq}}\Rightarrow\text{s }\geq\text{ }\frac{\text{u}\left(1-\text{t}\right)\left(1-\text{v}\right)-\text{v}}{\left(1-\text{v}\right)\left(1+\text{u}\right)}$**
14. Note also the special case $\text{a}=\frac{\text{y}_{\text{eq}}}{\text{x}_{\text{eq}}}>0\text{ }\Rightarrow\text{s}\geq\frac{\text{u}\left(1-\text{t}\right)\left(1-\text{v}\right)-\text{v}\frac{\text{y}_{\text{eq}}}{\text{x}_{\text{eq}}}}{\left(1-\text{v}\right)\left(\frac{\text{y}_{\text{eq}}}{\text{x}_{\text{eq}}}+\text{u}\right)}$
