# Supplementary material for: Home and away- the evolutionary dynamics of homing endonucleases
Source: BMC Evol Biol. 2011 Nov 4;11:324. doi: 10.1186/1471-2148-11-324 (PMC3229294; doi:10.1186/1471-2148-11-324)
Supplement: Additional file 4 — Proof S2 - Proof of analytical bound 2. Proof of analytical bound 2. [file 1471-2148-11-324-S4.DOCX]

**Additional file 4**

Proof S2- Proof of analytical bound 2

Theorem 2: **$\forall\text{a>0,az}_{\text{eq}}>\text{x}_{\text{eq}},\text{ z}_{\text{eq}}>\text{b}\Rightarrow\text{t }<\text{ as}+\frac{\text{v}}{\text{b}}$**

Proof:

1. At equilibrium: **$\text{y }=\text{ }\left(1-\text{v}\right)\left[\frac{\left(1-\text{s}\right)\text{y}}{\text{W}}+\frac{\text{u}\left(1-\text{s}-\text{t}\right)\text{z}}{\text{W}}\right]$**
2. It is biologically true that: **$\text{u}>0,\text{ s}+\text{t}<1$**
3. **$\text{y }=\text{ }0\Rightarrow\text{z }=\text{ }0$**
4. **$\text{y}\neq0,\text{z }\neq\text{ }0\Rightarrow\text{W}>\text{ }\left(1-\text{v}\right)\left(1-\text{s}\right)$**
5. **$1-\text{sy}-\left(\text{s}+\text{t}\right)\text{z }>\text{ }\left(1-\text{v}\right)\left(1-\text{s}\right)$**
6. **$\text{tz }<\text{ 1}-\text{sy}-\text{sz}-\left(1-\text{v}\right)\left(1-\text{s}\right)$**
7. **$\text{tz }<\text{ 1}-\text{sy}-\text{sz}-\left(1-\text{v}\right)\left(1-\text{s}\right)-\text{sx}+\text{sx}$**
8. **$\text{tz }<\text{ 1}-\text{s}-\left(1-\text{v}\right)\left(1-\text{s}\right)+\text{sx}$**
9. **$\text{tz }<\text{ v}-\text{vs}+\text{sx }<\text{ v}+\text{sx}$**
10. **$\text{t }<\text{ }\frac{\left(\text{v}+\text{sx}\right)}{\text{z}}$**
11. **$\text{az}_{\text{eq}}>\text{x}_{\text{eq}},\text{ z}_{\text{eq}}>\text{b}\Rightarrow\text{t }\leq\text{ as}+\frac{\text{v}}{\text{b}}$** **QED**
12. In particular **$\text{z }>\text{ }0.5\Rightarrow\text{t }\leq\text{ s }+\text{2v}$**
13. Note also the special case $\text{a= z}_{\text{eq}}>0\Rightarrow\text{t }\leq\text{ }\frac{\text{v}+\left(1-\text{z}_{\text{eq}}\right)\text{s}}{\text{z}_{\text{eq}}}$
