# Supplementary material for: Home and away- the evolutionary dynamics of homing endonucleases
Source: BMC Evol Biol. 2011 Nov 4;11:324. doi: 10.1186/1471-2148-11-324 (PMC3229294; doi:10.1186/1471-2148-11-324)
Supplement: Additional file 5 — Proof S3 - Proof of analytical bound 3. Proof of analytical bound 3. [file 1471-2148-11-324-S5.DOCX]

**Additional file 5**

Proof S3- Proof of analytical bound 3

Theorem 3: **$\text{z}_{\text{eq}}<\text{ }\frac{\left(\text{s}+\text{t}+\frac{\text{2v}}{\text{u}}\right)}{\text{hm}\left(1-\text{v}\right)\left(1-\text{u}\right)\left(1-\text{s}-\text{t}\right)}$**

Lemma 3.1: **$\text{x}_{\text{eq}}>\text{ }\frac{\text{u}}{2}$**

Proof of Lemma 3.1:

1. at equilibrium **$\begin{matrix} \text{z }=\text{ }\frac{\left(1-\text{v}\right)\left(1-\text{u}\right)\left(1-\text{s}-\text{t}\right)\text{z}}{\text{W}} \\ \cdot\left(1+\frac{\text{hm}\left[\text{x}+\text{v}\left(\text{W}-\text{x}\right)\right]}{\text{W}}\right) \end{matrix}$**
2. If z=0 then the theorem is self evident
3. **$\begin{matrix} \text{z }\neq\text{ }0\Rightarrow\text{W}^{2}=\left(1-\text{v}\right)\left(1-\text{u}\right)\left(1-\text{s}-\text{t}\right) \\ \cdot\left(\text{W}+\text{hm}\left[\text{x}+\text{v}\left(\text{W}-\text{x}\right)\right]\right) \end{matrix}$**
4. **$\begin{matrix} \text{W}^{2}-\left(1-\text{v}\right)\left(1-\text{u}\right)\left(1-\text{s}-\text{t}\right)\text{W }=\text{ } \\ \left(1-\text{v}\right)\left(1-\text{u}\right)\left(1-\text{s}-\text{t}\right)\text{hm}\left[\text{x}+\text{v}\left(\text{W}-\text{x}\right)\right] \end{matrix}$**
5. **$\begin{matrix} \text{W}^{2}-\left(1-\text{v}\right)\left(1-\text{u}\right)\left(1-\text{s}-\text{t}\right)\text{W} \\ -\text{ }\left(1-\text{v}\right)\left(1-\text{u}\right)\left(1-\text{s}-\text{t}\right)\text{v}\left(\text{W}-\text{x}\right)=\text{ } \\ \left(1-\text{v}\right)\left(1-\text{u}\right)\left(1-\text{s}-\text{t}\right)\text{hmx} \end{matrix}$**
6. **$\begin{matrix} \text{x }=\text{ }\frac{\text{W}^{2}-\left(1-\text{v}\right)\left(1-\text{u}\right)\left(1-\text{s}-\text{t}\right)\text{W}}{\left(1-\text{v}\right)\left(1-\text{u}\right)\left(1-\text{s}-\text{t}\right)\text{hm}} \\ -\frac{\text{ }\left(1-\text{v}\right)\left(1-\text{u}\right)\left(1-\text{s}-\text{t}\right)\text{v}\left(\text{W}-\text{x}\right)}{\left(1-\text{v}\right)\left(1-\text{u}\right)\left(1-\text{s}-\text{t}\right)\text{hm}} \end{matrix}$**
7. **$\text{x }>\text{ }\frac{\text{W}^{2}-\left(1-\text{v}\right)\left(1-\text{u}\right)\left(1-\text{s}-\text{t}\right)\text{W}-\text{v}}{\left(1-\text{v}\right)\left(1-\text{u}\right)\left(1-\text{s}-\text{t}\right)\text{hm}}$**
8. **$\begin{matrix} \text{x }>\text{ }\frac{\text{W}-\left(1-\text{v}\right)\left(1-\text{u}\right)\left(1-\text{s}-\text{t}\right)-\frac{\text{v}}{\text{W}}}{\left(1-\text{v}\right)\left(1-\text{u}\right)\text{hm}} \\ \left(\text{Because W}>1-\text{s}-\text{t}\right) \end{matrix}$**
9. **$\text{x }>\text{ }\left(1-\text{s}-\text{t}\right)\left(\frac{1-\left(1-\text{v}\right)\left(1-\text{u}\right)-\frac{\text{v}}{\text{W}\left(1-\text{s}-\text{t}\right)}}{\left(1-\text{v}\right)\left(1-\text{u}\right)\text{hm}}\right)$**
10. **$\text{x }>\text{ }\left(1-\text{s}-\text{t}\right)\left(\frac{\text{u}+\text{v}-\text{uv}-\text{ }\frac{\text{v}}{\text{W}\left(1-\text{s}-\text{t}\right)}}{\left(1-\text{v}\right)\left(1-\text{u}\right)\text{hm}}\right)$**
11. It is biologically plausible that **$\text{s}+\text{t}<0.25, \text{ 3v}<\text{u}<\frac{2}{9}$**
12. **$\begin{matrix} \left(1-\text{s}-\text{t}\right)^{2}>\text{ }\frac{1}{2-\text{u}}\Rightarrow\text{W}\left(1-\text{s}-\text{t}\right)\text{ }>\text{ }\frac{1}{2-\text{u}} \\ \Rightarrow\left(2-\text{u}\right)\text{W}\left(1-\text{s}-\text{t}\right)\text{ }>\text{ 1}\Rightarrow2-\text{u }>\text{ }\frac{1}{\text{W}\left(1-\text{s}-\text{t}\right)} \\ \Rightarrow1-\text{u}-\frac{1}{\text{W}\left(1-\text{s}-\text{t}\right)}\text{ }>\text{ }-1\Rightarrow\text{v}-\text{uv}-\text{ }\frac{\text{v}}{\text{W}\left(1-\text{s}-\text{t}\right)}\text{ }>\text{ }-\text{v} \end{matrix}$**
13. **$\text{x }>\text{ }\frac{\left(1-\text{s}-\text{t}\right)\left(\text{u}-\text{v}\right)}{\left(1-\text{v}\right)\left(1-\text{u}\right)\text{hm}}$**
14. **$\text{x }>\text{ }\left(1-\text{s}-\text{t}\right)\left(\text{u}-\text{v}\right)$**
15. **$\text{x}>\frac{\text{u}}{2}$** (using the above assumptions **$\text{s}+\text{t}<0.25, \text{3v}<\text{u}$**) **QED Lemma** 3.1

Proof of theorem 3:

1. At equilibrium****
2. **$\begin{matrix} \text{xW}^{2}=\text{ }\left[\text{x}+\text{v}\left(\text{W}-\text{x}\right)\right] \\ \cdot\text{W}\left(1-\frac{\text{hm}\left(1-\text{v}\right)\left(1-\text{u}\right)\left(1-\text{s}-\text{t}\right)\text{z}}{\text{W}}\right) \end{matrix}$**
3. **$\begin{matrix} \text{hm}\left(1-\text{v}\right)\left(1-\text{u}\right)\left(1-\text{s}-\text{t}\right)\text{z}\left[\text{x}+\text{v}\left(\text{W}-\text{x}\right)\right]\text{ }= \\ \text{ }\left[\text{x}+\text{v}\left(\text{W}-\text{x}\right)\right]\text{W}-\text{ xW}^{2} \end{matrix}$**
4. **$\text{z }=\text{ W}\frac{\left[\text{x}+\text{v}\left(\text{W}-\text{x}\right)-\text{xW}\right]}{\text{hm}\left(1-\text{v}\right)\left(1-\text{u}\right)\left(1-\text{s}-\text{t}\right)\left[\text{x}+\text{v}\left(\text{W}-\text{x}\right)\right]}$**
5. **$\text{z }<\text{ W}\left[\frac{\text{x}+\text{v}\left(\text{W}-\text{x}\right)-\text{xW}}{\text{hm}\left(1-\text{v}\right)\left(1-\text{u}\right)\left(1-\text{s}-\text{t}\right)\text{x}}\right]$**
6. **$\begin{matrix} \text{z }<\text{ }\frac{\text{W}\left(1-\text{W}\right)}{\text{hm}\left(1-\text{v}\right)\left(1-\text{u}\right)\left(1-\text{s}-\text{t}\right)}\text{ } \\ +\text{ }\frac{\text{Wv}\left(\text{W}-\text{x}\right)}{\text{hm}\left(1-\text{v}\right)\left(1-\text{u}\right)\left(1-\text{s}-\text{t}\right)\text{x}} \end{matrix}$**
7. **$\begin{matrix} \text{z }<\text{ }\frac{\text{W}\left(1-\text{W}\right)}{\text{hm}\left(1-\text{v}\right)\left(1-\text{u}\right)\left(1-\text{s}-\text{t}\right)} \\ \text{ }+\text{ }\frac{\text{v}}{\text{hm}\left(1-\text{v}\right)\left(1-\text{u}\right)\left(1-\text{s}-\text{t}\right)\text{x}} \end{matrix}$**
8. We notice that **$\begin{matrix} \text{W}\left(1-\text{W}\right)\text{ }<\text{ 1}-\text{W }=\text{ } \\ \text{sy}+\left(\text{s}+\text{t}\right)\text{z }=\text{ s}\left(\text{y}+\text{z}\right)+\text{tz }<\text{ s}+\text{t} \end{matrix}$**
9. **$\text{z }<\text{ }\left(\frac{\text{s}+\text{t}+\frac{\text{v}}{\text{x}}}{\text{hm}\left(1-\text{v}\right)\left(1-\text{u}\right)\left(1-\text{s}-\text{t}\right)}\right)$**
10. **$\text{z}_{\text{eq}}<\text{ }\left(\frac{\text{s}+\text{t}+\frac{\text{2v}}{\text{u}}}{\text{hm}\left(1-\text{v}\right)\left(1-\text{u}\right)\left(1-\text{s}-\text{t}\right)}\right)\text{ }\left(\text{using Lemma }3.1\right)$** **QED**

Supplementary proof 3a:

Theorem 3.a: **$\text{hm}>\frac{\text{s}+\text{t}}{\left(1-\text{v}\right)\left(1-\text{u}\right)}$**

1. **$\text{x }=\text{ }\frac{\text{x}+\text{v}\left(\text{W}-\text{x}\right)}{\text{W}}\cdot\left[1-\text{hm}\left(1-\text{v}\right)\left(1-\text{u}\right)\left(1-\text{s}-\text{t}\right)\frac{\text{z}}{\text{W}}\right]$**
2. **$\begin{matrix} \text{x}+\text{v}\left(\text{W}-\text{x}\right)\text{ }>\frac{\text{x}+\text{v}\left(\text{W}-\text{x}\right)}{\text{W}} \\ \cdot\left[1-\text{hm}\left(1-\text{v}\right)\left(1-\text{u}\right)\left(1-\text{s}-\text{t}\right)\frac{\text{z}}{\text{W}}\right] \end{matrix}$**
3. **$\begin{matrix} \text{x }>\text{ }\frac{\text{u}}{\text{2 }}\left(\text{Lemma 3.1}\right)\text{ }>\text{ }0 \\ \Rightarrow\text{W}^{2}>\text{ W}-\text{ hm}\left(1-\text{v}\right)\left(1-\text{u}\right)\left(1-\text{s}-\text{t}\right)\text{z} \end{matrix}$**
4. **$\text{hm}\left(1-\text{v}\right)\left(1-\text{u}\right)\left(1-\text{s}-\text{t}\right)\text{z }>\text{ W }-\text{ W}^{2}$**
5. **$\text{hm }>\text{ }\frac{\text{W}\left(1-\text{W}\right)}{\left(1-\text{v}\right)\left(1-\text{u}\right)\left(1-\text{s}-\text{t}\right)\text{z}}$**
6. **$\text{hm }>\text{ }\left[1-\text{sy}-\left(\text{s}+\text{t}\right)\text{z}\right]\frac{\left[\text{sy}+\left(\text{s}+\text{t}\right)\text{z}\right]}{\left(1-\text{v}\right)\left(1-\text{u}\right)\left(1-\text{s}-\text{t}\right)\text{z}}$**
7. **$\text{hm }>\text{ }\frac{\left(1-\text{s}-\text{t}\right)\left(\text{s}+\text{t}\right)\text{z}}{\left(1-\text{v}\right)\left(1-\text{u}\right)\left(1-\text{s}-\text{t}\right)\text{z}}$**
8. **$\text{hm }>\text{ }\frac{\text{s}+\text{t}}{\left(1-\text{v}\right)\left(1-\text{u}\right)}$QED**

Supplementary proof 3b:

Theorem 3b:

$$\begin{matrix} \text{az}_{\text{eq}}>\text{y}_{\text{eq}},\text{ z}_{\text{eq}}>\text{b} \\ \Rightarrow\text{hm}<\text{ }\frac{\left(\text{a}+1\right)\text{s}+\text{t}+\frac{\text{2v}}{\text{bu}}}{\left(1-\text{v}\right)\left(1-\text{u}\right)\left(1-\text{s}-\text{t}\right)} \end{matrix}$$

1. From supplementary proof 3, paragraph 8 in the proof of the main theorem: **$\begin{matrix} \text{z }<\text{ }\frac{\text{W}\left(1-\text{W}\right)}{\text{hm}\left(1-\text{v}\right)\left(1-\text{u}\right)\left(1-\text{s}-\text{t}\right)} \\ \text{ }+\text{ }\frac{\text{v}}{\text{hm}\left(1-\text{v}\right)\left(1-\text{u}\right)\left(1-\text{s}-\text{t}\right)\text{x}} \end{matrix}$**
2. Using lemma 3.1 we get **$\text{z }<\text{ }\frac{1-\text{W}-\frac{\text{2v}}{\text{u}}}{\text{hm}\left(1-\text{v}\right)\left(1-\text{u}\right)\left(1-\text{s}-\text{t}\right)}$**
3. **$\text{hm }<\text{ }\frac{\text{sy}+\left(\text{s}+\text{t}\right)\text{z}-\frac{\text{2v}}{\text{u}}}{\text{zhm}\left(1-\text{v}\right)\left(1-\text{u}\right)\left(1-\text{s}-\text{t}\right)}$**
4. **$\begin{matrix} \text{az}_{\text{eq}}>\text{y}_{\text{eq}},\text{ z}_{\text{eq}}>\text{b} \\ \Rightarrow\text{hm}<\text{ }\frac{\left(\text{a}+1\right)\text{s}+\text{t}+\frac{\text{2v}}{\text{bu}}}{\left(1-\text{v}\right)\left(1-\text{u}\right)\left(1-\text{s}-\text{t}\right)} \end{matrix}$** **QED**

In particular **$\text{z}_{\text{eq}}>0.5\Rightarrow\text{hm }\leq\text{ }\frac{\text{2s}+\text{t}+\frac{\text{4v}}{\text{u}}}{\left(1-\text{v}\right)\left(1-\text{u}\right)\left(1-\text{s}-\text{t}\right)}$**

In addition,
