# Supplementary figures and images for: Home and away- the evolutionary dynamics of homing endonucleases
Source: BMC Evol Biol. 2011 Nov 4;11:324. doi: 10.1186/1471-2148-11-324 (PMC3229294; doi:10.1186/1471-2148-11-324)

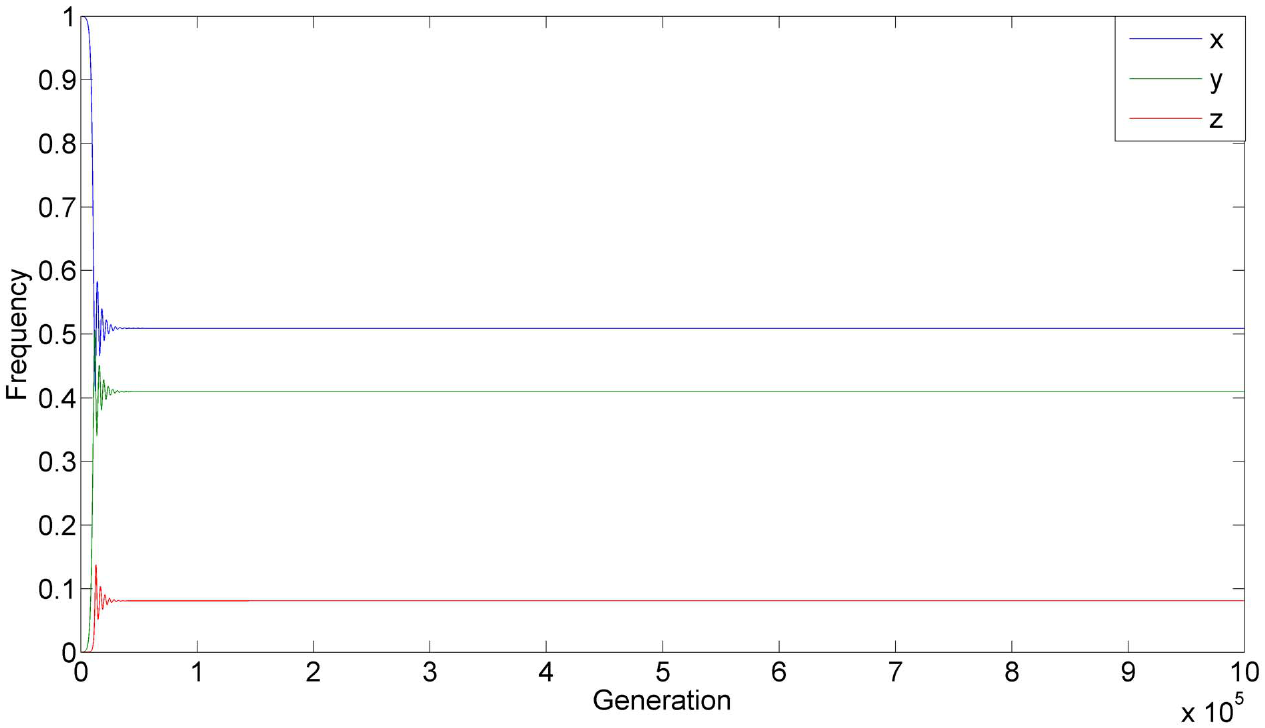

Supplement: Additional file 6 — Figure S1 - Examples of Homing endonuclease persistence in Yahara's model. Examples of Homing endonuclease persistence in Yahara's model obtained using the parameters: α = 0.989, β=α, r = 0.0123, u = 10-4, v = 10-6 and the initial frequency (in Yahara's notation): x = 0.9999, y = 10-4, z = 0. Iterating it for one million generations in Yahara's model, we get a stable equilibrium (determined using numeric computations of the eigenvalues) with frequencies X ≅ 0.51, y ≅ 0.41, z ≅ 0.081. [file 1471-2148-11-324-S6.TIFF]

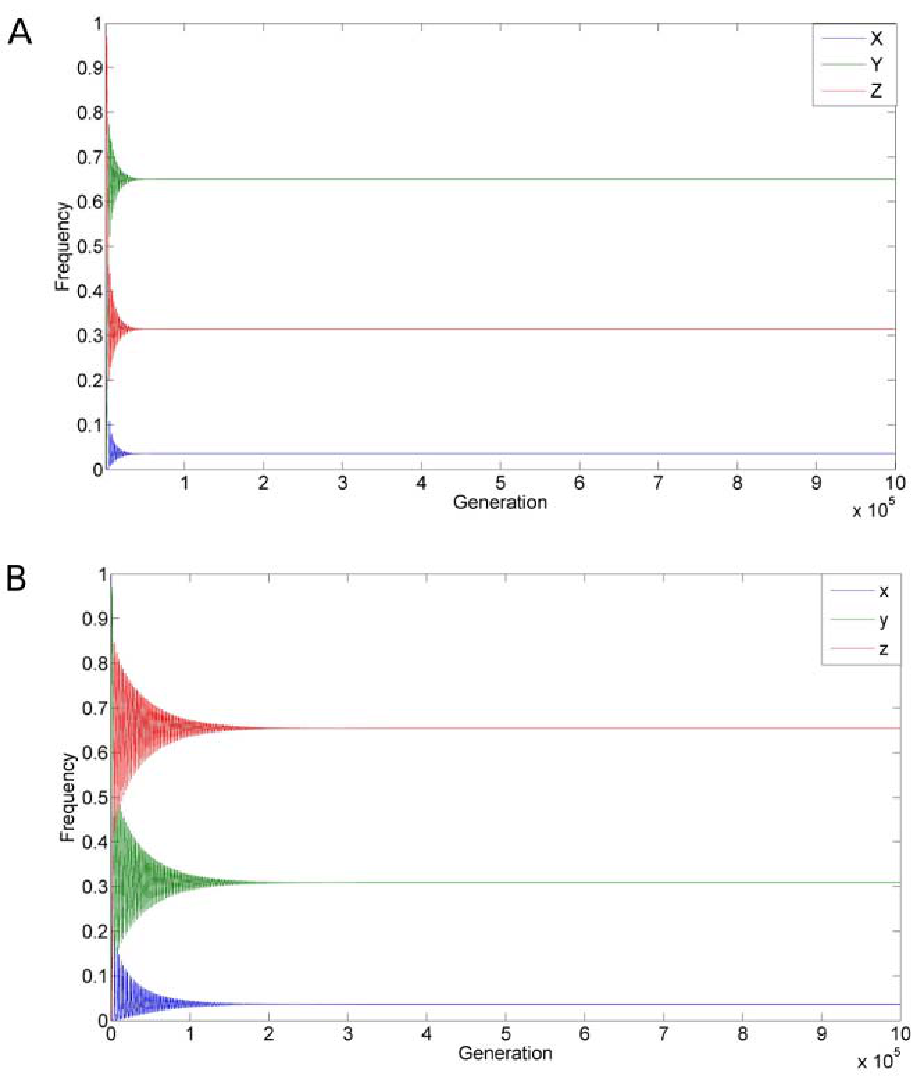

Supplement: Additional file 7 — Figure S2 - Examples of Homing endonuclease persistence at a high frequency. Examples of Homing endonuclease persistence at a high frequency in both ours and Yahara's models. Initial frequencies are the same as in Additional file 6. A) Stable equilibrium, using our model, with final z frequency of 0.314 obtained using the parameters: s = 0.01, t = 0.001, hm = 0.0323, u = 10-4 and v = 10-6. B) Stable equilibrium, using Yahara's model, with final z frequency of 0.655 obtained using parameters that are analogous the the parameters in A (as explained in Additional file 1, Table S1). [file 1471-2148-11-324-S7.TIFF]

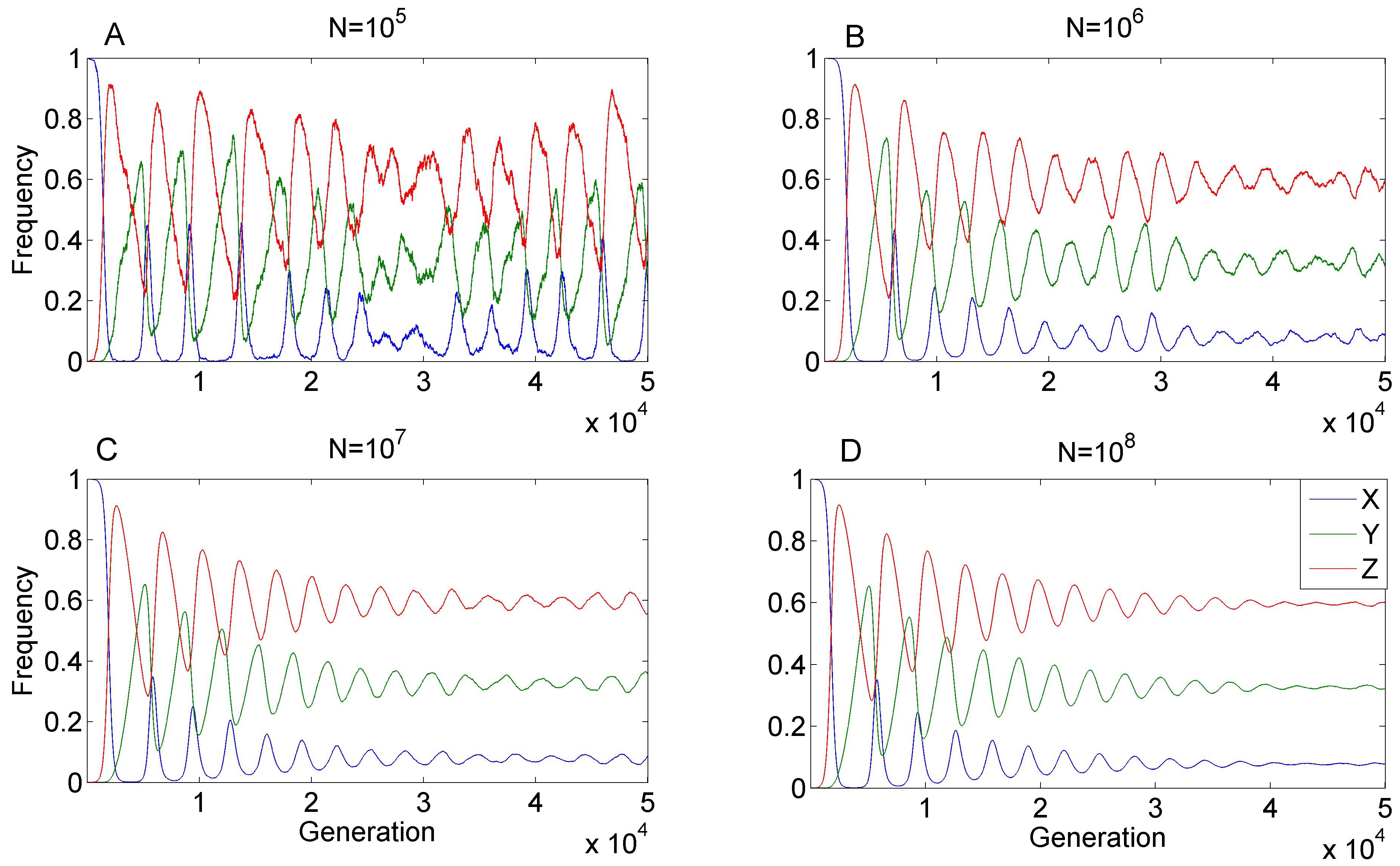

Supplement: Additional file 8 — Figure S3 - Example of model predictions for finite populations. Example of model predictions for finite populations. Random noise was added to our deterministic model as expected from binomial sampling in a population of size N = 105, 106, 107, 108 (A-D, respectively). The parameters used result in a stable equilibrium in the deterministic model: s = 0.01, t = 0.001, hm = 0.0165, u = 10-4 and v = 10-6. [file 1471-2148-11-324-S8.JPEG]
